# Discovery of Novel Alphacoronaviruses in European Rodents and Shrews

**DOI:** 10.3390/v8030084

**Published:** 2016-03-18

**Authors:** Theocharis Tsoleridis, Okechukwu Onianwa, Emma Horncastle, Emma Dayman, Miaoran Zhu, Taechasit Danjittrong, Marta Wachtl, Jerzy M. Behnke, Sarah Chapman, Victoria Strong, Phillipa Dobbs, Jonathan K. Ball, Rachael E. Tarlinton, C. Patrick McClure

**Affiliations:** 1 School of Life Sciences, University of Nottingham, Nottingham NG7 2UH, UK; stxtt6@exmail.nottingham.ac.uk (T.T.); nixoo@exmail.nottingham.ac.uk (O.O.); stxerh@nottingham.ac.uk (E.H.); stxed3@nottingham.ac.uk (E.D.); msxmz@nottingham.ac.uk (M.Z.); taechasit@live.com (T.D.); mzymw3@nottingham.ac.uk (M.W.); J.Behnke@nottingham.ac.uk (J.M.B.); patrick.mcclure@nottingham.ac.uk (C.P.M.); 2 Twycross Zoo, Burton Road, Atherstone, Warwickshire CV9 3PX, UK; sarah.chapman@twycrosszoo.org (S.C.); ntxvjs@exmail.nottingham.ac.uk (V.S.); phillipa.dobbs@twycrosszoo.org (P.D.); 3 School of Veterinary Medicine and Science, University of Nottingham, Sutton Bonington Campus, Loughborough LE12 5RD, UK; rachael.tarlinton@nottingham.ac.uk

**Keywords:** coronaviruses, rodents, shrews, alphacoronavirus

## Abstract

Eight hundred and thirteen European rodents and shrews encompassing seven different species were screened for alphacoronaviruses using PCR detection. Novel alphacoronaviruses were detected in the species *Rattus norvegicus*, *Microtus agrestis, Sorex araneus* and *Myodes glareolus.* These, together with the recently described Lucheng virus found in China, form a distinct rodent/shrew-specific clade within the coronavirus phylogeny. Across a highly conserved region of the viral polymerase gene, the new members of this clade were up to 22% dissimilar at the nucleotide level to the previously described Lucheng virus. As such they might represent distinct species of alphacoronaviruses. These data greatly extend our knowledge of wildlife reservoirs of alphacoronaviruses.

## 1. Introduction

Coronaviruses (CoVs) are important pathogens affecting both humans and animals. The first CoV discovery was made in the 1930s [1]. In humans, CoVs are associated with respiratory disease, ranging from mild upper respiratory tract symptoms, to those that are more severe and potentially fatal, associated with infection of the lower-respiratory tract. CoVs are also known to infect mammals and birds, among which they have been associated with enteric and respiratory diseases as well as hepatitis and neurological disorders [2]. The recent emergence of severe acute respiratory syndrome (SARS) and Middle East respiratory syndrome (MERS) in humans and porcine epidemic diarrhoea virus (PEDV) in pigs has highlighted the epizootic and zoonotic risk that these viruses pose (reviewed in [3,4]). It is very likely that there are novel unrecognized CoVs circulating in animals that pose a cross-species transmission risk.

Bats harbour a plethora of CoV species, some of which are thought to be the initial source of recent spillover events such as SARS, MERS and PEDV [3,4]. However, the recent discovery of new genetically distinct CoVs in rodents in China—Lucheng Rn rat CoV, Longquan A mouse CoV (LAMV) and Longquan Rl rat CoV (LRLV) [5], as well as a novel CoV in the *Rattus norvegicus* species, named China Rattus CoV (ChRCoV) HKU24 [6]—has highlighted that rodents may harbour unidentified CoVs with zoonotic or epizoonotic potential. The rodent family includes more than 2000 species [7] and collectively these harbour over 60 known human and livestock infectious diseases [8]. For rodents that live in close proximity to humans or domestic animals there is an increased risk of cross-species transmission following exposure to rodent carcasses, faeces, urine and parasites or, in the case of pigs, through direct consumption of infected carcasses [9,10]. To date, with the exception of murine hepatitis virus (MHV), there have been few reports of CoVs in European rodents. Here we report the discovery of novel CoVs in wild rodents and shrews in Europe.

## 2. Materials and Methods 

**Figure 1:**
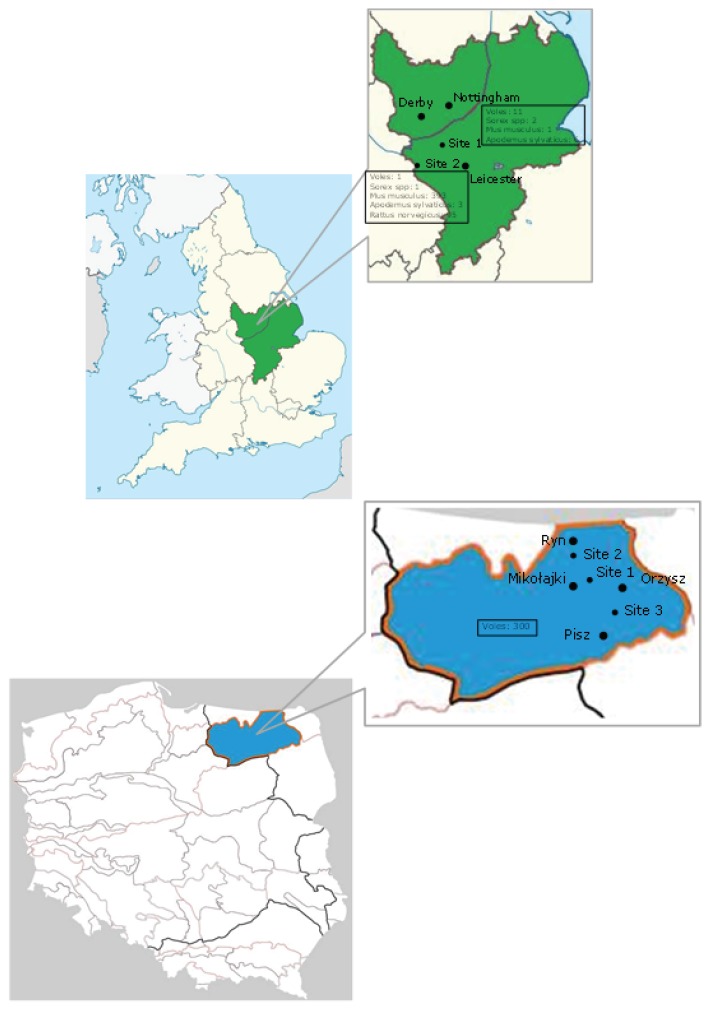
(**A**) Geographical map of the East Midlands region of the United Kingdom. Twenty samples were collected from Site 1 (52°49'13.0" N 1°15'01.9" W), four of which were positive for coronaviruses. Four hundred and ninety three samples were collected from Site 2 (52.6528° N, 1.5297° W), six of which were positive for coronaviruses; (**B**) Geographical map of the Mazury Lake District region of NE Poland. A total of 300 samples were collected from three sites (Site 1: 53°47.7 N 21° 39.6 W, Site 2: 53°53.5 N 21° 32.4 W, Site 3: 53°42.3 N 21° 48.8 W), one of which was positive for coronaviruses.

In total, 899 tissue samples from 813 wild rodents and shrews from the East Midlands region of the United Kingdom and the Mazury Lake District region of Poland (Figure 1, Table 1) were collected either as part of routine pest control, as a consequence of predation (*Felis catus*) or for other studies [11]. The samples from the United Kingdom were collected between 2008 and 2015 and the study was approved by the University of Nottingham Internal Ethics Committee (Nottingham, UK). The study for the Polish samples was approved by the Ethics Commission for Experiments on Animals of the M. Nencki Institute of Experimental Biology of the Polish Academy of Sciences (Warszawa, Poland). The rodent species included: house mouse (*Mus musculus)*, brown rat (*Rattus norvegicus)*, field vole *(Microtus agrestis),* bank vole (*Myodes glareolus*), wood mouse (*Apodemus sylvaticus*) and shrew (*Sorexaraneus)*. A necropsy was performed and tissue samples were stored at −70 °C in ethanol (Polish liver specimens), at −70 °C in RNAlater (Sigma Aldrich, St. Louis, MO, USA; UK liver and gut specimens) or at −20 °C without additives (UK liver specimens collected in 2008).

Total RNA, from approximately one cubic millimetre sections of liver or intestinal tissue samples, was extracted using the GenElute™ Mammalian Total RNA Miniprep Kit (Sigma Aldrich) and used as template in reverse-transcription PCRs using in-house designed primers targeting the rodent *glyceraldehyde 3-phosphate dehydrogenase* (*GAPDH*) gene (F: 5’-CCATCTTCCAGGAGCGAGA-3’, R: 5’-GCCTGCTTCACCACCTTCT-3’), *cytochrome b* gene [12] or a previously published primer set targeting a conserved region of the *ORF1ab* CoV polymerase gene [13]. Extra sequence for *ORF1ab* was acquired by using an in-house primer targeting an upstream conserved region (F: 5’-AATCTTAAGTATGCTATTAGTGG-3’) in combination with the previously described reverse primer [13]. PCR products of expected size were subject to Sanger sequencing (Source Bio Science, Nottingham, UK) and sequence similarity to Genbank database sequences was determined using BLASTn. Coronavirus *ORF1ab* and rodent *cytochrome b* reference sequence sets were downloaded from Genbank and used alongside sequences obtained in this study for phylogenetic analysis using the Molecular Evolutionary Genetics Analysis version 6 (MEGA6) software [14]. Codon-constrained nucleotide sequences were aligned using ClustalW and maximum likelihood phylogenetic trees (utilising a GRT with invariant sites (G+I) model of evolution) were generated, with robustness assessed using bootstrap resampling (1000 pseudoreplicates). CoV sequences generated in this study have been deposited in the Genbank database under accession numbers KU739070-KU739074.

## 3. Results and Discussion

**Figure 2:**
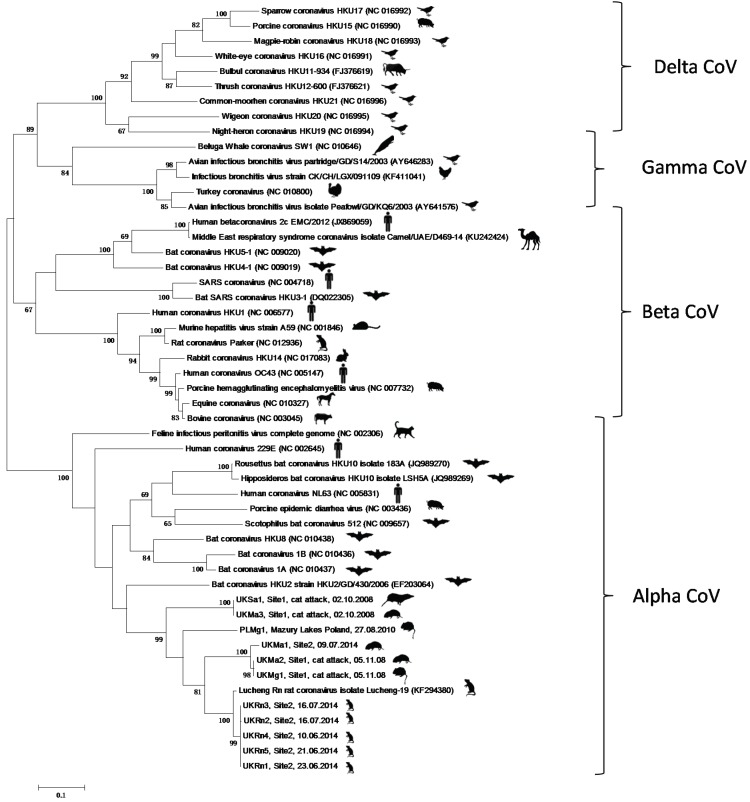
Maximum likelihood phylogenetic analysis of coronavirus partial *ORF1ab* gene sequences, corresponding to positions 8331-8960 on the Lucheng Rn isolate (Genbank Accession number KF294380. Novel coronavirus sequences obtained from UK-resident *Rattus norvegicus* (UK*Rn*), *Microtus Agrestis* (UK*Ma*), *Sorex araneus* (UK*Sa*) and Poland-resident (PL*Mg*) or UK-resident (UK*Mg*) were analysed alongside reference sequences representing the four different coronavirus genera. Reference sequences are indicated by their Genbank Accession Number. Branch lengths are drawn to scale: the bar indicates 0.10 nucleotide substitutions per site. Numbers above individual branches indicate the percentage that that branch was found in 1000 bootstrap replicates; only percentages >60 are shown.

**Figure 3:**
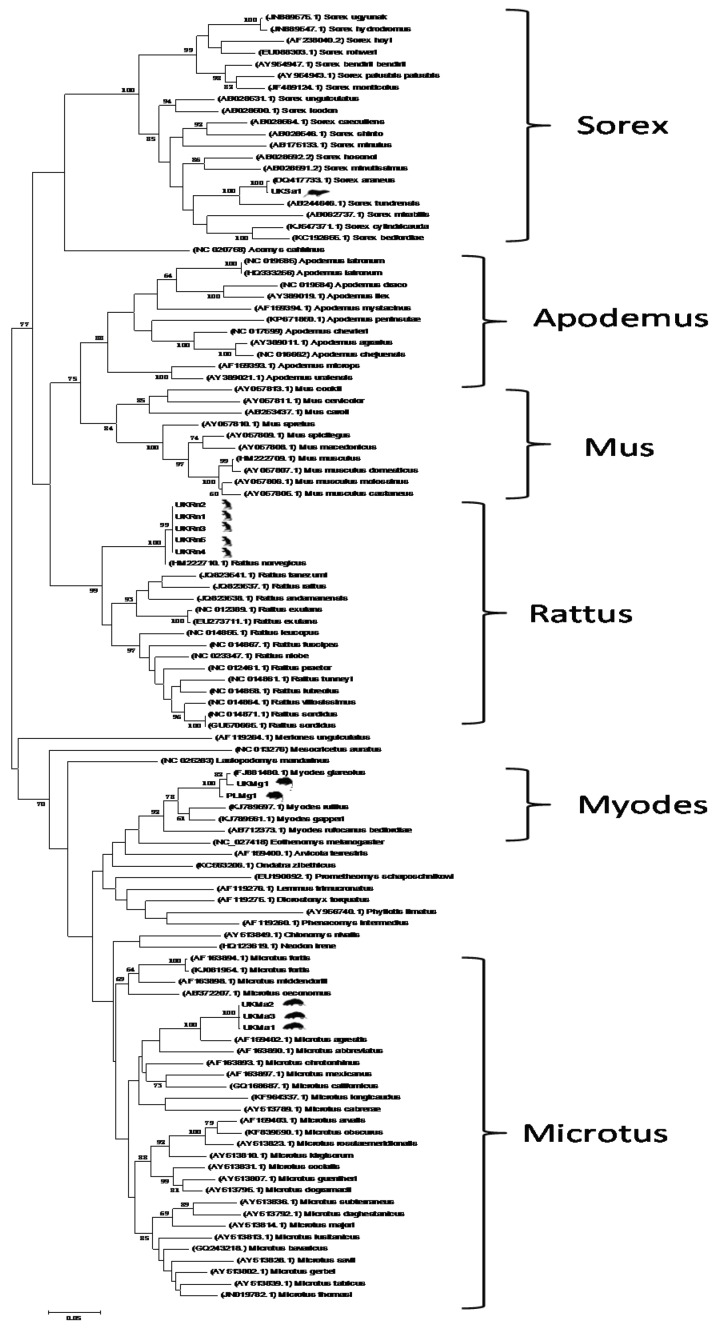
Maximum likelihood phylogenetic analysis of partial rodent *cytochrome b* nucleotide sequences. Host *cytochrome b* sequences obtained from CoV positive UK-resident *Rattus norvegicus* (UK*Rn*), *Microtus Agrestis* (UK*Ma*), *Sorex araneus* (UK*Sa*) and Poland-resident (PL*Mg*) or UK-resident (UK*Mg*) were analysed alongside reference sequences representing rodent and shrew *cytochrome b* sequences. Reference sequences are indicated by their Genbank Accession Number. Branch lengths are drawn to scale: the bar indicates 0.05 nucleotide substitutions per site. Numbers above individual branches indicate the percentage that that branch was found in 1000 bootstrap replicates; only percentages >60 are shown.

Eleven of the 813 rodents and shrews tested were found to be positive for CoVs (Table 1). Five positives were found in *Rattus norvegicus*, three in *Microtus agrestis*, one in *Sorex araneus* and two in *Myodes glareolus*. The phylogenetic analysis (Figure 2) showed that the new rodent/shrew CoVs, together with the Lucheng CoV reference strain formed a single clade (with 99% bootstrap support) in the *Alphacoronavirus* genus. Within this cluster one lineage contained a single sequence that was present in five separate samples (UK*Rn*1-5) obtained from *Rattus norvegicus,* which were most related to the Lucheng CoV (100% bootstrap support). A separate cluster with 100% bootstrap support contained three sequences obtained from *Microtus agrestis* (UK*Ma*1&2) and *Myodes glareolus* (UK*Mg*1). A third branch contained a sequence obtained from *Myodes glareolus* (PL*Mg*1) living in the Mazury lakes region of Poland and a further distinct sequence obtained from *Sorex araneus* (UK*Sa*1) and *Microtus agrestis* (UK*Ma*3) (99% bootstrap support). Detailed analysis of the sample archive, including details of the day and method of capture revealed that samples UK*Sa*1 and UK*Ma*3, which were identical across the region of *ORF1a1b* analysed, were not only obtained from the same geographical area but also captured by the same predator (*Felis catus*) on the same day. This was also true for samples UK*Ma*2 and UK*Mg*1. Therefore, we are left with three plausible explanations for the CoV phylogeny observed. Either the same or similar rodent coronaviruses were co-circulating and freely infecting different rodent species living in the same geographical area at the time of sampling; cross-species transmission of coronaviruses has been described previously [15,16,17,18,19]. An alternative explanation is that these carcasses were cross-contaminated during or after predation with coronavirus present in either animal or through predator-contamination from a different infected animal altogether that was not sampled. We do not consider that this result was due to contamination during tissue processing as new sterile dissection equipment was used for each carcass and this effect was observed between animals that were not processed consecutively. The *cytochrome b* sequence data (Figure 3) clearly showed that the specimens were of different species (arguing against sample mix-up). Importantly, re-extraction of the original rodent tissues gave identical results (data not shown), which strongly suggests that contamination did not occur during nucleic acid extraction or PCR preparation. Finally, it is also possible that these animals were infected with distinct strains of coronavirus but the PCR primers, which were designed to target highly conserved regions of the *ORF1ab* gene, were unable to discriminate between highly related viruses. The inability of *ORF1ab* PCR sequencing to resolve different isolates was evident in recent analyses of bat CoVs [20,21]. Sequencing additional regions of the genome, as has been performed previously with bat CoVs [22], together with analysis of larger numbers of rodents, will help elucidate the overall diversity and distribution of alphacoronaviruses in European rodents and shrews. Interestingly, the samples UK*Sa*1, UK*Ma*2&3 and UK*Mg*1 were killed by the same predator in a relatively limited sample set (Site 1, Figure 1), suggesting coronavirus pathogenesis may have increased susceptibility to predation in these animals. Whether or not coronavirus infection might render rodents more susceptible to predation needs further investigation.

The novel UK*Rn*1-5*,* UK*Ma*1*,* UK*Ma2*&UK*Mg*1*,* UK*Sa*1*/*UK*Ma*3 and PL*Mg*1 sequences were 97.6%, 83.8%, 84.6%, 78.4% and 81.8% similar and 98.6%, 96.2%, 96.2%, 91.9% and 94.3% similar to the corresponding region of the Lucheng *Rn* CoV, at the nucleotide and amino acid level, respectively (Table 2). 

**Figure 4:**
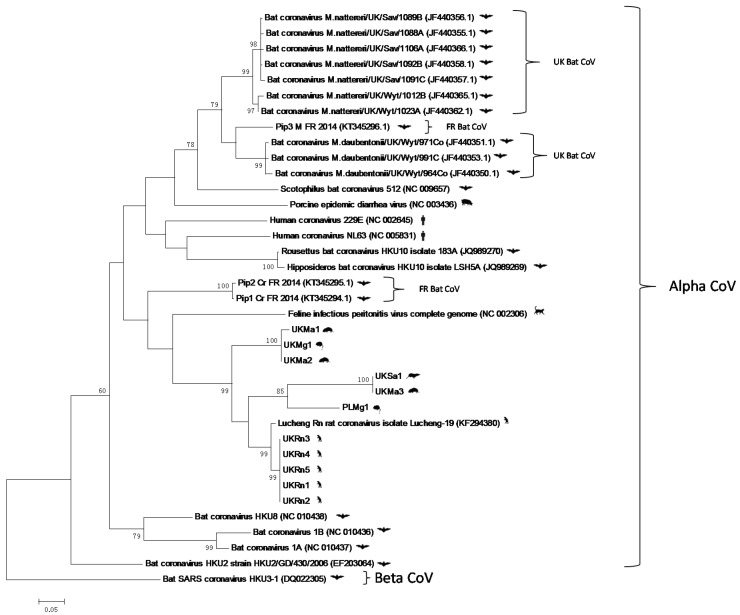
**(Overleaf):** Maximum likelihood phylogenetic analysis (rooted using the severe acute respiratory syndrome (SARS) bat HKU3 betacoronavirus as outgroup) of alphacoronavirus partial (394bp) *ORF1ab* gene sequences, corresponding to positions 8559-8952 on the Lucheng Rn isolate (Genbank Accession number KF294380. Novel coronavirus sequences obtained from UK-resident *Rattus norvegicus* (UK*Rn*), *Microtus Agrestis* (UK*Ma*), *Sorex araneus* (UK*Sa*) and Poland-resident (PL*Mg*) or UK-resident (UK*Mg*) were analysed alongside bat coronaviruses that were recently discovered in the United Kingdom (GenBank Accession numbers JF440350–JF440366) and France (KT345294–KT345296) and other reference sequences representing the *Alphacoronavirus* genus. CoV sequences obtained from studies of bats performed in UK and France are highlighted. Reference sequences are indicated by their Genbank Accession Number. Branch lengths are drawn to scale: the bar indicates 0.05 nucleotide substitutions per site. Numbers above individual branches indicate the percentage that that branch was found in 1000 bootstrap replicates; only percentages >60 are shown.

Many animal and human CoV spillover events can be ultimately traced back to a bat reservoir [23,24,25]. Therefore it is important to determine whether or not there is any evidence to suggest that the rodent CoVs described here are related to those circulating in mainland European/UK bats [21,26]. Although the sequences described in the bat analyses represented a shorter fragment of the *ORF1ab* gene, we were able to perform phylogenetic analyses with the novel rodent CoV sequence described here, and these comparisons showed that there was no evidence, based on phylogenetic clustering that the UK/European rodents were directly related to those present in bats (Figure 4). The nearest relative was FIPV, which infects cats, although the rodent/shrew and cat sequences did fall within a larger cluster that contained coronaviruses isolated from pipistrelle bats. Therefore, it is highly unlikely that the novel rodent/shrew CoVs represent a recent and direct epizootic event from bats. However, more wide scale analyses of CoV infection of European mammal populations needs to be performed to fully understand their evolutionary history.

## 4. Conclusions 

This study has shown the first evidence of alphacoronaviruses present in European rodents and shrews demonstrating that rodent and shrew coronaviruses sampled to date, from worldwide locations, form a discreet clade within this genus. CoV infection of rodents and shrews appears both geographically and temporally widespread and therefore these mammals may pose a threat for cross-species transmission to humans and/or other animals. Further characterization is required for better understanding of their genetic diversity, host range and cellular receptors.

## Figures and Tables

**Table 1:** List of animal species, location, organ, specimen tally and number of CoV positive samples.

Species	Location	Organ	Number	CoV Positives
*Mus musculus*	United Kingdom	Liver	394	0
		Gut	58	
*Rattus norvegicus*	United Kingdom	Liver	95	5 ^‡^
		Gut	28	
*Microtus agrestis*	United Kingdom	Liver	11	3
*Myodes glareolus*	United Kingdom	Liver	1	1
	Poland	Liver	300	1
*Sorex araneus*	United Kingdom	Liver	3	1
*Apodemus sylvaticus*	United Kingdom	Liver	9	0
				
	**Total animals**		**813**	
	**Total samples**		**899**	

^‡ ^ Two animals were positive in liver and gut, one animal was positive in the gut only and two animals had only a liver sample available.

**Table 2:** Pairwise percentage nucleotide (upper quadrant) and deduced amino acid (lower quadrant) similarities of the novel rodent and shrew Coronavirus (CoVs) and the Lucheng Rn CoV across a region of the *ORF1ab* gene corresponding to positions 8331-8960 on the Lucheng Rn isolate (Genbank Accession number KF294380). Comparisons were made using unique nucleotide sequences. Sequence identifiers are the same as those shown in Figure 2.

	Pairwise Percentage Similarity					
	Lucheng_*Rn*	UK*Rn*1-5	UK Ma1	UK*Ma*2/UK*Mg*1	UK*Sa*1/UK*Ma*3	PL*Mg*1
Lucheng_*Rn*		97.6	83.8	84.6	78.4	81.8
UK*Rn*1-5	98.6		83.7	84.6	77.8	81.3
UK*Ma*1	96.2	95.7		97.6	76.7	79.4
UK*Ma*2/UK*Mg*1	96.2	95.7	100		76.2	80.3
UK*Sa*1/UK*Ma*3	91.9	91.9	90.5	90.5		79
PL*Mg*1	94.3	93.8	93.8	93.8	89	
